# Polycystic ovary syndrome in familial partial lipodystrophy type 2 (FPLD2): basic and clinical aspects

**DOI:** 10.1080/19491034.2018.1509659

**Published:** 2018-10-03

**Authors:** Alessandra Gambineri, Laura Zanotti

**Affiliations:** Endocrinology Unit, Department of Medical and Surgical Sciences, St Orsola-Malpighi Hospital, Alma Mater University of Bologna, Bologna, Italy

**Keywords:** PCOS, FPLD2, severe insulin resistance, adipose tissue, lipodystrophy

## Abstract

Polycystic ovary syndrome (PCOS) is a common disorder with a high phenotypic variability. Frequently, it is associated with a mild to moderate insulin resistance (IR) caused by an interaction between polygenic diathesis and the environment. However, PCOS may be a complication of an underlying syndrome of severe IR such as insulin receptor autoantibodies, mutations in the insulin receptor or in the signalling pathway downstream from the insulin receptor or, most frequently, a defect in function or in the development of the subcutaneous adipose tissue. Such conditions are clinically characterized by lipodystrophy. Lipodystrophy in some cases is produced by a single-gene defect. In our experience, PCOS secondary to a missense mutation in the LMNA gene, known as familial partial lipodystrophy type 2 (FPLD2), is the most frequent form of PCOS secondary to severe IR due to genetically determined lipodystrophy. These forms should be identified as they benefit from tailored therapies.

## Introduction

Polycystic ovary syndrome (PCOS) is the most common endocrinopathy of women in reproductive age. Although the view of PCOS has gradually emerged as a largely heterogeneous disease with long-term health consequences including metabolic disorders and an increased cardiovascular risk, the diagnosis of this syndrome is still based on the association between hyperandrogenism and ovarian dysfunctions. This is defined by the presence of oligo-amenorrhea and chronic anovulation or polycystic ovarian morphology-PCOm at ultrasound.

In 1990, an expert conference sponsored by the National Institute of Health (NIH) established that the major criteria to diagnose PCOS included hyperandrogenism (determined by the presence of hirsutism and/or excess blood total testosterone) associated with oligo-amenorrhea and chronic anovulation, provided that all other well-known disorders characterized by androgen excess have been ruled out a priori [1]. In 2003, an expert conference in Rotterdam added a third criterion, based on the morphological appearance of the ovaries by ultrasonography-PCOm [2]. Intriguingly, the Rotterdam panel decided that PCOS could be defined when at least two major features were present, whatever their combination. The Rotterdam criteria therefore produced a great number of different possible phenotypes of PCOS, characterized by the presence or the absence of a hyperandrogenic state.

A few years later, the Androgen Excess and PCOS society (AEPCOS) [3] emphasized the importance of hyperandrogenism as a primary criterion to define PCOS, taking into account that metabolic alterations, chiefly insulin resistance (IR), diabetes, dyslipidemia, and non-alcoholic fatty liver disease-NAFLD are predominantly associated with the hyperandrogenic phenotype of PCOS [4].

Over time, the need to improve the evaluation of the diagnostic criteria of PCOS, as well as the clinical and therapeutic management of PCOS patients became increasingly evident. In 2012, a document from the Expert Panel at the NIH (US) suggested a list of actions aimed at improving the diagnosis and management of PCOS. This included the need to: i) define specific biological and clinical markers using a phenotype-biological approach; ii) better define the causes, predictors, and long-term health consequences of PCOS; and iii) improve the knowledge of specific phenotypes of PCOS with the final aim of ameliorating prevention and treatment strategies based on individual needs [5–7].

Among the specific phenotypes of PCOS, the rare forms secondary to severe IR states emerged. These forms include those due to Familial Partial Lipodystrophy Type 2 (FPLD2), that are the object of this review. It is important for clinicians to be able to identify the forms of PCOS that are secondary to severe IR states as they often harbour identifiable single-gene defects and benefit from tailored therapies.

## Clinical and biochemical characteristics that suggest PCOS in the context of severe insulin resistance states

The most common form of IR, which is very prevalent in both lean and overweight/obese PCOS women as a metabolic complication, is due to an interaction between polygenic diathesis and the environment, with poorly defined contributory genes and with a severity variable [4]. However, a subgroup of PCOS is characterized by an extreme form of IR that is the primary event. Patients with severe IR states typically have either profound compensatory hyperinsulinemia or diabetes which require high levels (over 200 units per day) of exogenous insulin for glycaemic control. Although no formal criteria for the biochemical diagnosis of severe IR exists, there is general consensus that the following conditions are indicators: a fasting insulin value above 20.9µU/mL and/or a peak insulin value on oral glucose tolerance testing above 209 µU/mL when diabetes is absent and a body mass index (BMI) below 30kg/m^2^; or an exogenous insulin requirement of above 3 U/kg per day when diabetes with absolute insulin deficiency is present in association with a BMI below 30kg/m^2^ [8].

However, the biochemical diagnosis of severe IR becomes difficult in patients with partial β-cell compensation and/or in subjects with a BMI above 30kg/m^2^ due to background obesity related IR, glucotoxicity, impaired islet function, and mixtures of endogenous and exogenous insulin in the blood. In this condition, IR should be interpreted in the light of clinical history and physical signs, although high c-peptide levels can help in the diagnosis of severe IR in insulin-treated patients. A physical sign, which is very frequent in severe IR states, is acanthosis nigricans. This cutaneous hyperpigmentation is typically located on the intertriginous surface and neck, and in the most severe cases on perioral, periocular, and buccal regions or even on planar surfaces. Other physical signs of severe IR states which are not always present, are unusual severe combined dyslipidemia (high triglycerides and low HDL-cholesterol levels), sometimes complicated by eruptive xanthomata and episodes of acute pancreatitis, unusually severe NAFLD and early-onset hypertension [8].

## Severe insulin resistance and lipodystrophy

Severe IR states can be subcategorized by the type of defect that leads to IR. These defects include insulin receptor autoantibodies, mutations in the insulin receptor or in the signalling pathway downstream from the insulin receptor and, most frequently, in a defect in function or in the development of the subcutaneous adipose tissue. Such conditions are clinically characterized by lipodystrophy, which can be generalized or partial, acquired or congenital [8].

In the last few decades a combination of linkage analysis and candidate gene screening has led to the identification of a list of genes associated with congenital lipodystrophy, either generalized or partial, which encode essential proteins for normal fat tissue development and/or function in humans.

Based on the current knowledge, genes implicated in the pathogenesis of lipodystrophy can be categorized as being primarily involved in: i. the transcriptional regulation of adipocyte differentiation, such as peroxisome proliferator activated receptor y-PPARG, a member of the nuclear hormone receptor superfamily, and LMNA, encoding lamin A/C which is a nuclear envelope protein with many cellular roles including chromatin and transcription factor binding and organization of the nuclear membrane and cytoskeleton; ii. fatty acid uptake; iii. triacylglycerol (TAG) synthesis; iv. and lipid droplet formation, such as perilipin-PLIN1 which resides on the surface of the lipid droplet and plays a regulatory role in triglyceride mobilization [9]. The most frequent form of congenital lipodystrophy is the partial form due to missense mutations at the level of the LMNA gene, known as FPLD2. FPLD2 is phenotypically characterized by the loss of subcutaneous fat in arms, legs (more prominently in the forearms and calves than in the upper arms and thighs), a variable and progressive loss of subcutaneous fat from the anterior abdomen and chest, and an abnormal gain of fat in the face and neck. In addition, there is an abnormal increase in visceral fat in the abdomen. FPLD2, and congenital partial lipodystrophies in general, do not usually show clinical signs until puberty, when failure or abnormality of the natural pubertal accretion and changes in body shape unmask the underlying abnormality.

## PCOS secondary to lipodystrophy

Lipodystrophy in females is frequently accompanied by PCOS, and the main link between these two conditions is the severe IR state, and specifically, the ‘partial’ IR state, which means that some tissues are severely insulin resistant, particularly those involved in the metabolic effects of insulin. On the other hand, other tissues, such as the pituitary, adrenal, ovary, and to some extent the liver, maintain a level of insulin sensitivity and are thus exposed to the biological effects of exaggerated circulating insulin levels which compensate IR.

At the pituitary level, insulin increases the response of LH to GnRH, finally stimulating ovarian LH-mediated androgen production [10]. At the hepatic level, insulin inhibits the synthesis of the sex hormone binding globulin (SHBG), thereby increasing the amount of free androgens and consequently the peripheral androgen action. Insulin also inhibits the synthesis of the IGF binding protein (IGFBP)-1, leading to increased bioavailability of IGF-1 and -2, two important regulators of ovarian follicular maturation and androgen production from theca cells [10]. At the adrenal and ovarian levels, insulin increases androgen synthesis by stimulating cytochrome P450c17α activity, a key enzyme in the biosynthesis of ovarian and adrenal androgens which has both 17α-hydroxylase and 17,20-lyase activities. In addition, at the ovarian level, insulin contributes to anovulation directly by interfering with follicular development, causing premature follicular atresia and antral follicular arrest, and indirectly by affecting the effectiveness of gonadotropins in an abnormal intraovarian environment and by increasing intraovarian androgen levels with the subsequent collapse of small antral follicles into the ovarian stroma, leading to stromal hypertrophy and promoting the process of ovarian atresia [10]. Taken together, these effects lead to an increased synthesis of androgens by the adrenal and the ovarian thecal cells, an increased peripheral bioavailability of androgens, and the arrest of follicular growth in the ovary with subsequent anovulation, finally producing PCOS [10].

There may be other factors involved in linking lipodystrophy with PCOS, in particular lipotoxicity due to adipose tissue dysfunction, which may directly alter the oocyte quality in females through the production of free fatty acids (FFAs). Accordingly, both animal and human studies demonstrate that the accumulation of FFAs within the ovary is associated with endoplasmic reticulum stress, mitochondrial dysfunction of the oocytes, and ultimately apoptosis of the cumulus–oocyte complexes [11]. These data highlight various possible cellular mechanisms which may lead to impaired ovulation, reduced oocyte quality, and therefore to PCOS in lipodytrophic women. However, further studies are needed to corroborate these hypotheses.

## PCOS secondary to FPLD2

There are few reports in the literature describing the association of PCOS with FPLD2 [12–15].

In our experience, PCOS secondary to FPLD2 is the most frequent form of PCOS secondary to genetically determined lipodystrophy and is more common than supposed. In fact, 1% of PCOS patients referred to our clinic in the last 10 years have been found to have partial congenital lipodystrophy, the most common lipodystrophy being FPLD2 (0.75% of the entire population). In detail, of the 1200 patients visiting our out-patient clinic for symptoms of PCOS (oligo-amenorrhea, hirsutism, infertility), we found 18 cases with a partial lipodystrophic phenotype. Of these, twelve cases were affected by a partial congenital lipodystrophy; in detail, nine were affected by an heterozygous missense mutation in gene LMNA (Table 1), one case was affected by an heterozygous missense mutation in gene PPARG, and two cases were affected by an heterozygous missense mutation in gene PLIN1. It is possible that the link between FPLD2 and PCOS is due not only to the condition of severe IR and, probably, to the ovarian lipotoxicity due to FFA accumulation, but also to intrabdominal adipose tissue accumulation. In fact, visceral adipose tissue is an important site of androgen production, because of the expression of trigger enzymes, such as 3β-dehydrogenase and 17β-hydroxydehydrogenase [16]. The intra-adipose increased synthesis of androgens in association with a decrease in SHBG concentrations due to severe IR are responsible for a condition of ‘relative functional hyperandrogenism’ which may further affect ovarian function therefore contributing to the development of PCOS. In addition, it has been reported that visceral adipose tissue is characterized by a local low-grade inflammation, with a consequent increased production of cytokines, chemokines, and adipokines which are able to antagonize insulin functions, and with a decreased production of the natural insulin sensitizer adiponectin [17], which are all conditions that aggravate IR.

**Table 1.** Clinical and biochemical characteristics of a group of patients with PCOS secondary to FPLD2 visiting our out-patient clinic for the first time.

Pt	LMNA mutation	Main complaint	Age (years)	BMI (kg/m^2^)	T (ng/ml)	FGlu (mg/dl)	F Ins (mcU/ml)	H	AN	High Tg	NAFLD	DM	HBP	CV events	PCOm*
Pt 1	R482Q	Hirsutism	21	26	0.7	72	21.0	Y	Y	Y	N	N	N	**N**	**Y**
Pt 2	R482 Q	Amenorrhea	15	25	1.2	80	33.3	Y	Y	Y	Y	N	N	**N**	**Y**
Pt 3	R482W	Amenorrhea	22	23	0.3	83	26.8	Y	Y	Y	N	N	N	**N**	**N**
Pt 4	R482Q	Hirsutism	35	22	0.2	95	21.3	Y	Y	Y	N	N	N	**N**	**Y**
Pt 5	R482Q	Hirsutism	62	24	0.6	100	16.2	Y	Y	Y	**Y**	**Y**	**Y**	**Y**	**N**
Pt 6	R482Q	Hirsutism	59	26	0.8	101	94.0	Y	Y	Y	Y	Y	Y	**Y**	**Y**
Pt 7	R482Q	Hirsutism	31	26	0.5	92	30.2	Y	Y	Y	Y	N	N	**N**	**Y**
Pt 8	E202K	Amenorrhea	34	24	0.2	74	20.9	Y	Y	Y	Y	N	N	**N**	**Y**
Pt9	E202K	Hirsutism	16	22	0.8	83	21.6	Y	Y	Y	N	N	N	**N**	**Y**

Pt, patient; BMI, body mass index; T, testosterone; FGlu, Fasting glucose; FIns, Fasting insulin H, hirsutism; AN, acanthosis nigricans; Tg, triglycerides; NAFLD, non alcoholic fatty liver disease; DM, diabetes; HBP, hypertension; CV, cardiovascular events; Y, yes; N, no.

* PCOm was defined by the presence of a follicle number per ovary of ≥12 and/or an ovarian volume ≥10 ml

No substantial clinical differences among the form of PCOS secondary to FPLD2 and the other forms of PCOS secondary to partial congenital lipodystrophy were observed, nor among the form of PCOS secondary to FPLD2 and the forms of PCOS associated with severe IR without any identified genetic cause. Among the various biomarkers that could guide the clinician to suspect a form of PCOS secondary to FPLD2 we found of interest the quantification of the distribution of fat mass by dual energy X-ray absorptiometry (DXA), in particular the Fat Mass Ratio (FMR), calculated as the ratio between the percentage of trunk fat mass and the percentage of the lower-limb fat mass [18]. FMR in our cases was able to discriminate PCOS secondary to FPLD2 (1.7 ± 0.2, mean± SD) from the forms of PCOS associated with severe IR without any identified genetic cause (1.3 ± 0.1, mean± SD) and from controls (0.93 ± 0.10, mean± SD), whereas neither hypertriglyceridemia nor low adiponectin or leptin circulating levels had a high diagnostic accuracy (unpublished data).

The identification of the PCOS secondary to FPLD2 is important in clinical practice because these patients benefit from tailored therapies, including specific dietary management [19] as well as insulin sensitizers, particularly thiazolidinediones, which are PPARY agonists [15], and in some cases, by metreleptin therapy [20,21]. In addition, facial and neck lipoaspiration seems to be beneficial in these patients. We recently observed an improvement in metabolic alterations and a remission of the PCOS phenotype in patients treated by lipoaspiration of the middle third and of the lower third of the face and of the neck. Buffalo hump’s adipose tissue was also lipoaspirated (Table 2). These data need to be supported by further evidence.

**Table 2.** Clinical and biochemical parameters before and after one year from lipoaspiration.

	Patient 3		Patient 5	
	Before	After	Before	After
BMI (kg/m^2^)	**23.7**	**23.5**	**24.7**	**24.6**
Fasting glucose (mg/dL)	80	73	90	82
Fasting insulin (µU/mL)	16.6	9.3	20.7	10
Insulin peak at OGTT (µU/mL)	291	164	413	227
Menses	Amenorrhea	Eumenorrhea	Menopause	=
Hirsutism		Amelioration		Amelioration
Hypertension	No	No	Yes, uncontrolled	Yes, well controlled
Concomitant treatments	Metformin Pioglitazone	Metformin Pioglitazone	Metformin Rosuvastatin Candesartan	Metformin Rosuvastatin Candesartan

## Conclusions

Clinicians who deal with PCOS should be alerted to the forms of PCOS secondary to lipodystrophy, particularly to FPLD2, as they are not as uncommon as may be thought and respond to tailored therapies. The diagnosis of these forms remains predominantly clinical by examining patients in their underwear looking out for clinical hallmarks, supported by imaging biomarkers of alterations in body composition measured by DXA. Gene screening is necessary to corroborate the diagnosis but must be performed at the end of the diagnostic path.

Further studies are needed to describe the phenotypic features, diagnostic procedures, and potential therapeutic approaches to this secondary form of PCOS in more detail.
